# Experience in diagnosis and treatment of duodenal ulcer perforation in children

**DOI:** 10.1186/s12887-023-03957-8

**Published:** 2023-03-30

**Authors:** Qiulong Shen, Tingting Liu, Siwei Wang, Li Wang, Dayong Wang

**Affiliations:** 1 Department of Emergency Surgery, Beijing Children’s Hospital, Capital Medical University, National Center for Children’s Health, No.56 Nanlishi St, Xicheng District, Beijing, 100045 China; 2 Department of Ultrasound, Beijing Children’s Hospital, Capital Medical University, National Center for Children’s Health, No.56 Nanlishi St, Xicheng District, Beijing, 100045 China

**Keywords:** Duodenal ulcer, Duodenal perforation, Children

## Abstract

**Background:**

This study aims to summarize our experience in diagnosis and treatment of pediatric duodenal ulcer perforation in a National Center for Children’s Health.

**Methods:**

Fifty-two children with duodenal perforation hospitalized in Beijing Children’s Hospital Affiliated to Capital Medical University from January 2007 to December 2021 were retrospectively collected. According to the inclusion and exclusion criteria, patients with duodenal ulcer perforation were included in the group. They were divided into the surgery group and the conservative group according to whether they received surgery.

**Results:**

A total of 45 cases (35 males and 10 females) were included, with a median age of 13.0 (0.3–15.4) years. Forty cases (40/45, 88.9%) were over 6 years old, and 31 (31/45, 68.9%) were over 12 years old. Among the 45 cases, 32 cases (32/45, 71.1%) were examined for Helicobacter pylori (HP), and 25 (25/32, 78.1%) were positive. There were 13 cases in the surgery group and 32 cases in the conservative group, without a significant difference in age between the two groups (P = 0.625). All cases in the surgery group and the conservative group started with abdominal pain. The proportion of history time within 24 h in the two groups was 6/13 and 12/32 (P = 0.739), and the proportion of fever was 11/13 and 21/32 (P = 0.362). The proportion of pneumoperitoneum in the surgery group was higher than that in the conservative group (12/13 vs. 15/32, P = 0.013). The fasting days in the surgery group were shorter than those in the conservative group (7.7 ± 2.92 vs. 10.3 ± 2.78 days, P = 0.014). There was no significant difference in the total hospital stay (13.6 ± 5.60 vs14.8 ± 4.60 days, P = 0.531). The operation methods used in the surgery group were all simple sutures through laparotomy (9 cases) or laparoscopy (4 cases). All patients recovered smoothly after surgery.

**Conclusion:**

Duodenal ulcer perforation in children is more common in adolescents, and HP infection is the main cause. Conservative treatment is safe and feasible, but the fasting time is longer than the surgery group. A simple suture is the main management for the surgery group.

## Background

Duodenal ulcer perforation is one of the common acute abdominal diseases in adults, but it is relatively rare in children. At present, the relevant guidelines [1, 2] for peptic ulcer indicate that the conservative treatment of duodenal ulcer is restricted to a very small number of cases with limited perforation confirmed by water-soluble contrast agent examination. Most of them recommend surgical treatment, believing that delaying the operation may increase mortality. However, most studies referred to in the guidelines are adult studies, and whether they are applicable to children needs to be confirmed by further studies. Therefore, we summarize the experience in the diagnosis and treatment of pediatric duodenal ulcer perforation in a National Center for Children’s Health.

## Methods

This retrospective study included 52 patients diagnosed with duodenal perforation from January 2007 to December 2021 at Beijing Children’s Hospital Affiliated to Capital Medical University. The study adhered to the ethical principles of the Declaration of Helsinki and was approved by the Ethics Committee of Beijing Children’s Hospital (Number: 2022-E-129-R, 2022-6-8).

**Fig. 1:**
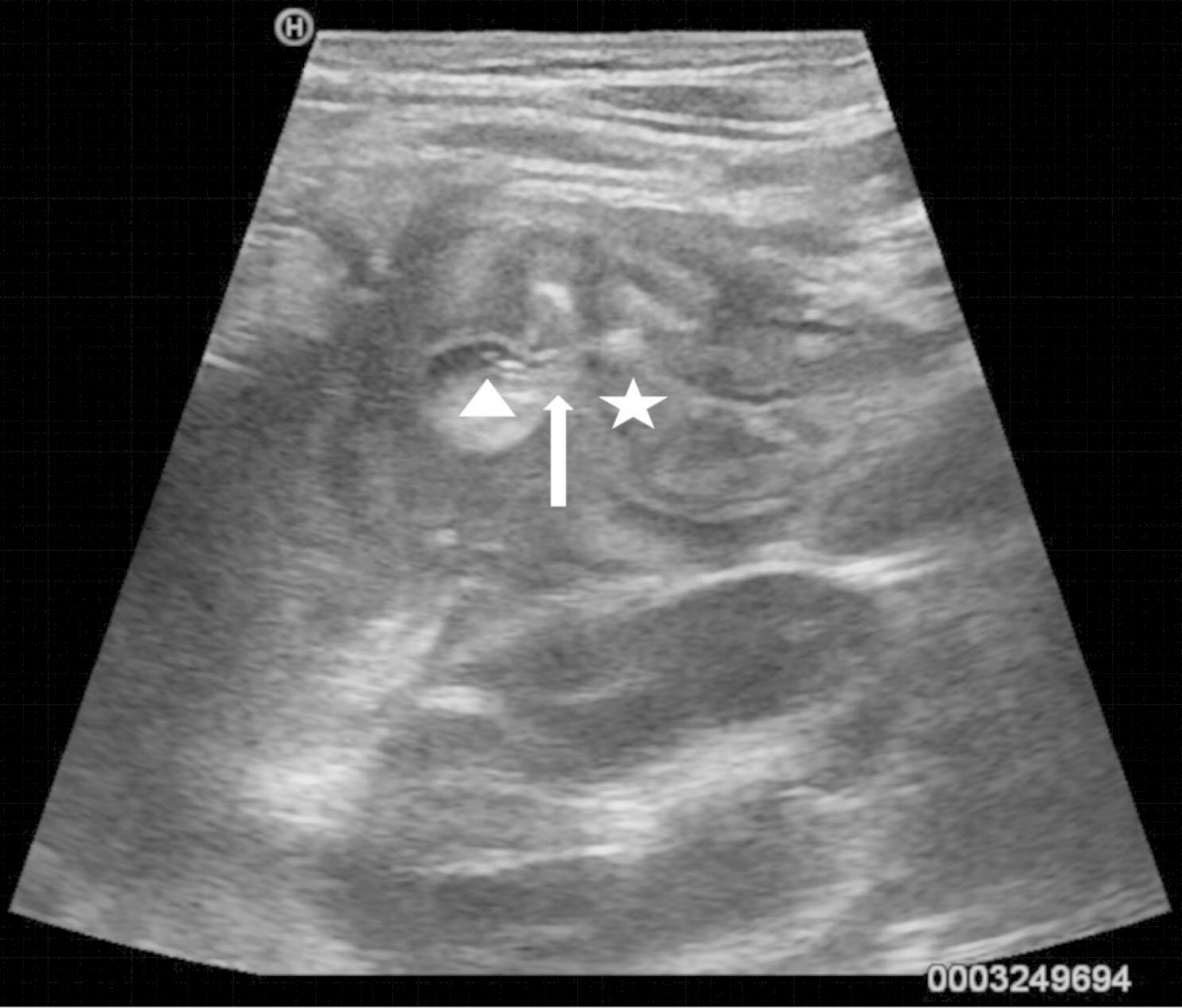
Sonogram of duodenal ulcer perforation. Sonogram shows gas and fluid accumulation around the duodenum(triangle), discontinuous duodenal intestinal wall(arrow). The star mark represents the duodenal cavity

According to the inclusion and exclusion criteria, the patients with duodenal ulcer perforation were included in the study. Inclusion criteria: patients with duodenal ulcer perforation diagnosed by imaging. All cases in this study were confirmed by ultrasonography (Fig. 1). Ultrasonographic manifestations include gas and fluid accumulation around the duodenum, discontinuous duodenal intestinal wall, surrounding swelling, free ascites in the abdominal cavity, and free gas in front of the liver [3]. Exclusion criteria: (1) duodenal perforation caused by trauma, foreign body, or iatrogenic causes; (2) patients with jejunal feeding tube.

The patients were then divided into the surgery group and the conservative group according to whether they received surgery. The clinical characteristics, including symptoms, family history, timing of onset of illness, radiological results, surgical procedures, fasting day, hospital stay and outcomes, were collected and compared between the two groups.

All patients received nil-per-os, decompression via nasogastric tube, intravenous hydration, antibiotics, and proton pump inhibitor (PPI) therapy. The criteria for oral feeding in the conservative group: (1) complete remission of symptoms; (2) no positive signs on physical examination; (3) no overflow of contrast medium in gastrointestinal contrast examination or no gas accumulation and effusion outside the duodenal wall in ultrasound examination. Surgery group: (1) complete remission of symptoms; (2) no positive signs on physical examination; (3) self-exhaust and defecate.

Statistical analysis was performed using IBM SPSS19.0. Categorical variables were analyzed with the χ2 test or Fisher’s exact test. Continuous variables with normal distributions are presented as the means ± standard deviations and were analyzed with Student’s t-test. Continuous variables with nonnormal distributions are presented as medians and ranges and were analyzed with the Mann-Whitney test. P < 0.05 (2-sided) was considered significant.

## Results

**Fig. 2:**
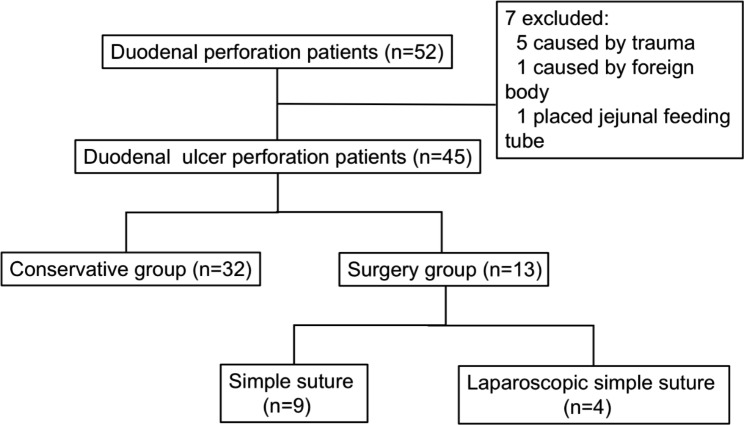
Flow Chart of Patient Enrollment

A total of 45 cases with duodenal ulcer perforation met the inclusion and exclusion criteria, including 35 males (35/45, 77.8%) and 10 females (10/45, 22.2%). Seven patients were excluded, including 5 caused by trauma, 1 caused by foreign body, and 1 placed jejunal feeding tube (Fig. 2). The median age at admission was 13.0 (0.3–15.4) years, with 40 cases (40/45, 88.9%) over 6 years old and 31 (31/45, 68.9%) over 12 years old. Of these cases, 32 (32/45, 71.1%) were examined for Helicobacter pylori (HP), and 25 (25/32, 78.1%) were positive. There were no differences in HP positivity between the conservative group (17/23) and the surgical group (8/9), P = 0.640. There were 13 cases without HP examination, of which 1 had a history of methylprednisolone use, the primary disease was cutaneous abdominal purpura, and the other 12 cases had unclear aetiology. Only one male child had a drinking history.

There were 13 cases in the surgery group and 32 cases in the conservative group. The median ages at admission in the surgery group and conservative group were 13.1 (1.0, 15.1) years old and 12.8 (0.3, 15.4) years old, respectively (P = 0.625). Abdominal pain was the first symptom in all cases. The proportions of history time within 24 h in the surgery group and conservative group were 6/13 and 12/32, respectively (P = 0.739). The proportion of vomiting and fever did not differ significantly between the two groups (vomiting, 10/13 vs. 24/32, P = 1.000; fever, 11/13 vs. 21/32, P = 0.362). All cases had signs of peritonitis. The proportions of total abdominal peritonitis in the surgery group and conservative group were 9/13 and 12/32, respectively (P = 0.098). The proportion of history of abdominal pain (presentation of abdominal pain besides that which led to the present hospitalization) did not differ significantly between the two groups (4/13 vs. 11/32, P = 1.000). Only 2 cases in the conservative group had a history of abdominal pain in their family member. The leukocytes in the two groups before treatments were (13.8 ± 4.52)×10^9^/L and (14.0 ± 6.47)×10^9^/L, respectively (P = 0.931). The C-reactive protein before treatments were 16.5 (8.75, 95) mg/dl and 10 (8, 62) mg/dl, respectively (P = 0.326). The proportion of pneumoperitoneum in the surgery group was higher than that in the conservative group (12/13 vs. 15/32, P = 0.013) (Table 1).

**Table 1:** General characteristics of surgery group and conservative group

	Surgery group (n = 13)	Conservation group (n = 32)	P value
**Gender: male(n)** **female(n)**	11 2	24 8	0.698
**Age(year)**	13.1(1.0, 15.1)	12.8(9.3, 15.4)	0.625
**Onset within 24 h(n)**	6	12	0.739
**Abdominal pain(n)**	13	32	-
**Vomiting(n)**	10	24	1.000
**Fever(n)**	11	21	0.362
**Total abdominal peritonitis (n)**	9	12	0.098
**Blood leukocytes (×10** ^**9**^ **/l)**	13.8 ± 4.52	14.0 ± 6.47	0.931
**X ray(free air) (n)**	12	15	0.013
**Fasting time(d)**	7.7 ± 2.92	10.3 ± 2.78	0.014
**Total hospital stay(d)**	13.6 ± 5.60	14.8 ± 4.60	0.531

In the surgery group, 11 cases were perforated at the duodenal bulb and 2 at the descending portion. The diameter of the perforation was 0.2-1.0 cm. Absorbable sutures were used for simple perforation repair, including 9 cases of laparotomy and 4 cases of laparoscopy. The characteristics of the surgery group are shown in Table 2.

**Table 2:** The characteristic of the surgery group

case	Sex/age(year)	X ray (free air)	HP	Location	Diameter (cm)	Surgical procedure	Complication
**1**	M/12.8	+	+	Bulb	0.3	Simple suture	No
**2**	M/13.9	+	+	Bulb	0.5	simple suture	No
**3**	M/13.3	+	+	Bulb	0.2	simple suture	No
**4**	M/13.8	+	+	Bulb	0.5	simple suture	No
**5**	F/1.0	-	/	Descending portion	0.8	simple suture	No
**6**	M/13.1	+	/	Bulb	0.3	simple suture	No
**7**	M/5.4	+	/	Descending portion	0.3	simple suture	No
**8**	M/14.9	+	+	Bulb	0.8	Laparoscopic simple suture	No
**9**	M/12.3	+	-	Bulb	1.0	Laparoscopic simple suture	No
**10**	M/13.4	+	+	Bulb	1.0	Laparoscopic simple suture	No
**11**	M/13.0	+	+	Bulb	0.4	Laparoscopic simple suture	No
**12**	F/11.2	+	/	Bulb	1.0	simple suture	Wound infection
**13**	M/15.1	+	+	Bulb	0.2	simple suture	No

*“/” represents that no relevant inspection has been carried out*

The fasting time of cases in the surgery group was much shorter than those in the conservative group (7.7 ± 2.92 vs. 10.3 ± 2.78 days, P = 0.014). However, the total hospital stay did not differ significantly between the two groups (13.6 ± 5.60 vs. 14.8 ± 4.60 days, P = 0.531). With a mean follow-up time of 0.8 (0.5-2) years after discharge, there were no postoperative complications observed with the exception of 1 case of postoperative wound infection in the surgery group. There was no recurrence of perforation in all cases.

## Discussion

The lifetime prevalence rate of peptic ulcer in the general population is 5–10%, with an annual incidence rate of 0.1–0.3% [4]. Peptic ulcer with perforation is usually classified into two groups, gastric and duodenal ulcer perforation. The incidence and degree of severity of duodenal ulcer perforation are lower than that of gastric ulcer perforation. Duodenal ulcer perforation occurs mainly in adults and rarely in children. The incidence of duodenal ulcer perforation in adults accounts for 2.8–4.3% of all peptic ulcers [5].

An adult study has reported that the number of men with duodenal ulcer perforation is almost 3 times that of women, which is similar to the findings of our study [6]. Moreover, the median age of males with duodenal ulcer perforation is also significantly lower than that of females. This condition is mainly due to a higher frequency of unhealthy habits in males, such as irregular diet, overeating, smoking, and drinking [7]. However, in our study, only 1 male adolescent had a clear drinking history, and the rest had no unhealthy habits.

As observed in our study, most cases with duodenal ulcer perforation were adolescents (over 12 years old), similar to the results reported in the literature [8]. All cases in our study had a history of acute abdominal pain, most of which were accompanied by vomiting, fever and peritonitis signs, but few cases had severe sepsis and septic shock which differs from ulcer perforation in adults. It may be related to the fact that most children have no underlying disease. Less than half of the cases in our study had a history of previous abdominal pain, and most had ulcer perforation at the onset of acute disease. We consider that is related to childrens’ inability to accurately describe abdominal discomfort.

The causes of duodenal ulcer mainly include HP infection, steroidal anti-inflammatory drugs, stress response, and gastrinoma [9–11]. HP infection is the most common cause of peptic ulcer and often has family aggregation. The HP infection rate in developing countries is as high as 30% [10, 12]. However, only 2 cases in our study had a history of familial HP infection. Another study reported that the most common cause of duodenal ulcer perforation in children was the use of dexamethasone [13]. Steroidal anti-inflammatory drugs can stimulate the secretion of gastric acid and pepsin, inhibit the secretion of gastric mucus, and lead to ulcers. But only 1 case in our study had a clear history of using steroidal anti-inflammatory drugs (glucocorticoids). This child had a history of abdominal purpura, in which the main manifestation was abdominal pain, thus masking the symptoms of ulcers and causing the presence of ulcers to be neglected.

The abdominal CT scan is considered the primary diagnostic method of duodenal ulcer perforation [14]. Suspicious CT scan findings include unexplained intraperitoneal fluid, pneumoperitoneum, bowel wall thickening, mesenteric fat streaking, and the presence of extraluminal water-soluble contrast. However, up to 12% of patients with perforations may have a normal CT scan [1]. CT scan has radiation hazards, so it is less used in children, and ultrasound examination is more widely used. Some studies have reported that abdominal ultrasound can also diagnose duodenal ulcer perforation when performed by a trained operator [1, 3]. Ultrasonographic manifestations include gas and fluid accumulation around the duodenum, discontinuous duodenal intestinal wall, surrounding swelling, free ascites in the abdominal cavity, and free gas in front of the liver. Our center’s experience also showed that ultrasonic diagnosis of duodenal ulcer perforation was accurate and reliable. All cases in our study were diagnosed by ultrasound. In addition, abdominal X-ray can be supplementary, showing free gas in the abdominal cavity. 60% of the cases in our study had a definite pneumoperitoneum performance. If there is no free gas on the images but duodenal ulcer perforation is still suspected, the absorbable contrast medium can also be used in upper gastrointestinal radiography to determine whether there is liquid overflow.

Regarding the treatment of ulcer perforation, the relatively recognized treatment is active surgical intervention, especially for patients over 70 years old [2] or those with immunosuppression [15]. Delaying the operation time may increase the mortality of patients with ulcer perforation. Simple suture is the most common surgical procedure

for perforation of duodenal ulcer. If the perforation diameter is large, omental tamponade repair [13, 16, 17] or distal gastric plus bulbar resection and gastrojejunostomy can be performed [18]. In addition, with the development of endoscopy, more and more endoscopic treatment and endoscopic combined surgery have been reported to treat duodenal ulcer perforation [19–21]. In our study, the diameters of perforation in the surgery group were all less than 1 cm. All patients underwent a simple suture, and the postoperative recovery was very well.

Although surgical treatment is advocated for duodenal ulcer perforation, conservative treatment is also safe and feasible for some patients. According to the Japanese guidelines for peptic ulcer [2], the criteria of conservative treatment includes onset within 24 h and when hungry, a stable condition without severe complication, the symptom of peritoneal irritation localized in the upper quadrant, and a small amount of ascites. A review on duodenal perforation in Sweden [14] proposed that conservative treatment was feasible for localized perforation with stable vital signs, but emergency surgical intervention was recommended if sepsis or septic shock was present. Pediatric sepsis is currently defined as the triad of fever, tachycardia, and vasodilation in addition to a change in mental status or prolonged capillary refill greater than 2 s [22]. In this group, there is no case of sepsis. All cases recovered smoothly, and there was no development of severe sepsis or septic shock [22]. In addition, the results of this study show that factors such as onset within 24 h or when hungry, and localized peritonitis are not necessary reference indicators for selecting surgical or conservative treatment. However, our study showed that the fasting time of patients with duodenal ulcer perforation in the conservative group was longer than in the surgery group. Patients in the conservative group were discharged once they started oral feeding, while patients in the surgery group was discharged after 2–3 days of observation after oral feeding. Ultimately, the two groups had no significant difference in the total hospital staying time.

Limitations in our study include the fact that this is a retrospective paper, and we were unable to pinpoint the criteria used to differentiate between patients who should undergo surgical treatment or conservative treatment. Another limitation is population bias, as our data is from a single-center unit. More patient cases from other centers would make this a more reliable study.

## Conclusions

In conclusion, duodenal ulcer perforation in children is more common in adolescents, and HP infection is the main cause. Conservative treatment is safe and feasible, but the fasting time is longer than that in the surgery group. Most of the operations are performed by simple suture.

## Data Availability

The data is available from the corresponding author on reasonable request.

## Abbreviations

HP: Helicobacter Pylori
CT: Computed Tomography
PPI: Proton Pump Inhibitor
